# Associations among Motor Competence, Physical Activity, Perceived Motor Competence, and Aerobic Fitness in 10–15-Year-Old Youth†

**DOI:** 10.3390/children11020260

**Published:** 2024-02-17

**Authors:** Dawn P. Coe, Emily M. Post, Eugene C. Fitzhugh, Jeffrey T. Fairbrother, E. Kipling Webster

**Affiliations:** 1 Department of Kinesiology, Recreation, and Sport Studies, The University of Tennessee, Knoxville, Knoxville, TN 37996, USA; fitzhugh@utk.edu (E.C.F.); kipwebster@utk.edu (E.K.W.); 2 Department Health & Sport Sciences, Otterbein University, Westerville, OH 43081, USA; post4@otterbein.edu; 3 School of Kinesiology, Auburn University, Auburn, AL 36849, USA; jtf0049@auburn.edu

**Keywords:** motor competence, perceived motor competence, fitness, physical activity, adolescent

## Abstract

(1) Background: The developmental model describes possible mechanisms that could impact the trajectory of children and adolescents’ health behaviors related to obesity; however, few data are available that support this model in the adolescent population. This study investigated the associations among motor competence (MC), moderate-to-vigorous physical activity (MVPA), perceived motor competence (PMC), and aerobic fitness in children and adolescents and the mediating and moderating effects of PMC, aerobic fitness, and weight status on the MC–MVPA relationship. (2) Methods: Participants included 47 adolescents (12.2 ± 1.6 y; 55% male) who completed the Bruininks–Oseretsky Test of Motor Proficiency, 2nd Edition (MC), Harter’s perceived self-competency questionnaire (PMC), and the PACER test (aerobic fitness) and whose MVPA was measured via accelerometry. The body mass index (BMI) was calculated from measured height and weight. (3) Results: There were positive correlations between MC and fitness [*r_s_*(47) = 0.469, *p* < 0.01], PMC and fitness [*r_s_*(47) = 0.682, *p* < 0.01], and PMC and MC [*r_s_*(47) = 0.416, *p* < 0.01]. There were no associations among MVPA and MC, PMC, or fitness (*p* > 0.05). There were inverse associations between BMI and both MVPA [*r_s_*(44) = −0.410, *p* < 0.01] and fitness [*r_s_*(47) = 0.295, *p* < 0.05]. The association between MC and MVPA was mediated by fitness (β = 0.3984; 95% CI (0.0564–0.7985)). (4) Conclusions: The associations among MC, PMC, and fitness highlight the critical role of MC in health and partially support the proposed developmental model concerning the relationships that exist among MC, MVPA, PMC, fitness, and BMI.

## 1. Introduction

Participation in regular physical activity is critical to youth development and is related to numerous health benefits including improved cardiometabolic health, physical fitness, and cognitive and mental wellness [1,2]. Physical activity behaviors and their benefits track from childhood through adolescence and eventually to adulthood [3,4]. Unfortunately, there has been a decline in the number of children and adolescents who meet the recommended 60 min per day of moderate-to-vigorous physical activity (MVPA) over the past few decades, which has negative implications for healthy youth development and subsequent adulthood behaviors [2,5,6,7]. The Physical Activity Guidelines for Americans focus specifically on MVPA throughout middle childhood and adolescence, rather than habitual physical activity, as it is related to better overall health outcomes [8]. Therefore, identifying factors related to MVPA participation and mechanisms for facilitating engagement in physical activity among children and adolescents is critical to fostering a physically active lifestyle.

Stodden et al. proposed a conceptual model emphasizing the importance of motor competence (MC), the ability to execute motor skills in a proficient manner, in facilitating engagement in physical activity [9,10]. Children who fail to develop the requisite fundamental motor skills as a foundation for MC may lead to less engagement in exercise, sport, and game participation, along with subsequent decreases in their engagement in a healthy amount of physical activity [8]. Specifically, poor fundamental motor skills may create a skill proficiency barrier that compromises the subsequent development of specialized motor skills and sports-specific skills [11,12]. This has been emphasized by De Meester and colleagues, who found that only 12% of older children with poor fundamental motor skills meet physical activity guidelines [13]. Children with poor MC may face increasing challenges in establishing and maintaining healthy activity levels that promote health-related physical fitness. Therefore, it is important to understand the relationship between MC and MVPA in youth.

The developmental model describes possible developmental mechanisms that could impact the trajectory of children and adolescents’ health behaviors related to obesity and the central role that MC and physical activity play in children’s health [10]. The model also postulates a dynamic and reciprocal relationship between MC and physical activity, whereas in late childhood, MC drives physical activity engagement. This relationship is further mediated by children’s health-related physical fitness and perceptions of competence throughout childhood. Evidence has supported a few of these pathways in early and middle childhood, but few have examined the applicability of this proposed framework in later childhood and adolescent age groups [14,15]. Of particular interest in this endeavor is an exploration in how the MC–MVPA relationship is potentially mediated by perceived motor competence (PMC), physical fitness, and weight status in adolescents.

A motivational factor that is related to participation in activity isPMC. PMC is an individual’s belief in their abilities across a range of different gross and fine motor skills [16]. PMC can either positively or negatively impact a child’s physical activity engagement, because children who perceive their motor skills to be better tend to persist in engaging in activities related to those skills and view themselves more positively, which motivates them to participate in physical activity, leading to higher levels of physical fitness [17]. Presumably, PMC may be more strongly related to physical activity in older children and adolescents due to their improved cognitive skills [18]. Multiple studies in the later childhood/adolescent age group have shown positive associations between PMC and MC, physical activity, and fitness, and a negative association between PMC and body mass index (BMI) [18,19]. However, additional studies in this age group are needed to provide sufficient support for the role PMC plays in the proposed developmental model. The majority of studies that found a significant mediating effect of PMC on the relationship between MC and physical activity largely included children in later childhood and adolescence [14,20,21,22]. Although these studies reported a mediating effect of PMC, additional evidence is needed to strengthen the applicability of this relationship in later childhood and adolescence [14].

The potential mediating effect of physical fitness on the MC–MVPA relationship also requires further examination [14]. Health-related physical fitness, indexed by aerobic capacity, muscular strength, muscular endurance, and flexibility, has been found to be related to both MC and PMC [23,24]. Children who are more physically active have been shown to have higher levels of health-related physical fitness and MC, whereas children who are less physically active have been shown to have lower levels of health-related physical fitness and MC [23,25]. Several studies have explored physical fitness as a mediating variable on the MC and physical activity relationship. Two studies have confirmed the mediating effect of physical fitness during middle childhood [21,26]. Cattuzzo et al. conducted a review of studies on the relationship between physical fitness and MC and identified aerobic fitness as the component that had the strongest association with MC [27]. As older children often participate in games or activities that require sustained engagement in physical activity behaviors, it makes sense that a higher competence level of motor coordination and motor abilities may bolster aerobic fitness in this older age group.

Weight status is generally considered the outcome of the developmental trajectory model outlined by Stodden and colleagues (2008) that is primarily influenced by MC and physical activity [10]. This factor is important because low levels of PMC, MC, and health-related physical fitness are thought to lead to obesity in youth [28]. Additionally, the inverse relationship between weight status and MC potentially creates a cycle in which continued deceleration in MC will perpetuate and magnify sedentary behaviors, leading to an increased overweight/obese weight status for many children [28,29]. Furthermore, it is plausible that children with a lower PMC will avoid physical activity, fail to develop adequate motor skills, and have more difficulty in maintaining a healthy body composition [10,28]. Inverse associations between weight status and MC, MVPA, PMC, and aerobic fitness have been established [15,18,19,30]. However, studies investigating the mediating and moderating roles of weight status on the MC–MVPA relationship are limited and further examination is warranted. Although the mediating and moderating effects of weight status deviate from the developmental model, the morphological constraints associated with obesity may logically impact engagement in MVPA, providing sound justification for this investigation.

The relationships among MC, physical activity, PMC, and aerobic fitness have been more extensively studied in young children compared to those in late childhood and adolescence. Specifically, research has identified positive relationships among MC, physical activity, PMC, and health-related physical fitness in early childhood (3–5 years old) and middle childhood (5–10 years old); research focusing on these variables during late childhood and adolescence (10–15 years old) is limited [10,14,15,17,31]. Therefore, the purpose of this study was to determine the associations among MC, MVPA, PMC, and aerobic fitness in late childhood and adolescence (10–15 years old) as proposed in the developmental model. Additionally, this study aims to investigate the possible mediating effects of PMC and aerobic fitness and the moderating effect of weight status on the MC–MVPA relationship.

## 2. Materials and Methods

### 2.1. Study Participants

Within a cross-sectional study design, participants included 47 youth (ages 10–15 years), who were recruited from two communities, located in east Tennessee and northwest Ohio. Participants were recruited through social media postings, flyer distribution, emails, and word of mouth. Exclusion criteria included any medical condition reported by a parent/guardian that would limit participation in physical activity. The investigator obtained written assent from each participant after reading a description of the study to the participants. Each participant signed an assent form after the investigator read the description of the study from the parental permission form. Written parental permission was then obtained for each participant by the parental guardian(s). The study protocol was approved by the university’s Institutional Review Board.

### 2.2. Study Design

Participants attended two separate sessions at either a research laboratory or a local gymnasium. At each site, identical protocols were used for all participants. Visit 1 was one hour in duration and Visit 2 was thirty min in duration. There was a seven-day time- period between Visit 1 and Visit 2 to reduce the probability of fatigue and to increase compliance for the 7-day accelerometer wear time protocol by returning the device in person. All activities were the same for all participants on Visit 1 and Visit 2; no randomization of assessments occurred. All assessments were completed by one of the co-authors (E.M.P.).

During Visit 1, after obtaining written parental permission and verbal participant assent, standing height, seated height, and weight were measured and BMI and maturity offset were calculated. Then, participants completed the Bruininks–Oseretsky Test of Motor Proficiency (BOT-2) and reported their PMC using one of the scales described below based on their specific age. After the completion of these assessments, participants were fitted with an accelerometer and parents/guardians and children were provided verbal instructions on appropriate usage. Visit 2 was scheduled at least one full week after Visit 1. Participants returned the accelerometer and completed the FitnessGram Physical Fitness Test Battery. In addition, each participant completed the same PMC questionnaire used during Visit 1.

#### 2.2.1. Bruininks–Oseretsky Test Analysis Test for Motor Proficiency

The participants completed the Bruininks–Oseretsky Test for Motor Proficiency, Second Edition [32]. BOT-2 measures fine and gross motor development and includes eight subtests (fine motor precision, fine motor integration, manual dexterity, body coordination, balance, running speed and agility, upper-limb coordination, and strength) consisting of a total of 53 items. The assessments were scored individually and combined to create four categorical standard scores, which included fine motor control (fine motor precision and fine motor integration), manual coordination (manual dexterity and upper limb coordination), body coordination (bilateral coordination and balance), and strength and agility (strength and running speed and agility). The four categorical values were compiled to create a total motor composite score and overall percentile using the BOT-2 normative reference tables. Research team members were trained with the BOT-2 Comprehensive Training video, which provides training on test administration, scoring, and reporting. Training also included the completion of 10–15 practice trials. Data integrity for the current study was further supported using reliability testing on a randomly selected sample of scores from approximately 10% of the participants. Interrater reliability was excellent (ICC = 0.99; 95% confidence interval = 0.99–1.00).

BOT-2 raw scores for each activity were used to determine scores and motor percentiles for four categories: fine manual coordination, manual coordination, body coordination, and strength and agility. Categorical scores were used to calculate the total motor composite score and total motor percentile following BOT-2 scoring procedures [32]. The total motor composite score was used in the statistical analysis. Percentiles were used to categorize each participant (10–13 years) for descriptive purposes.

#### 2.2.2. Perceived Motor Competence

Each participant completed a PMC assessment using the Perceived Competence Scale for Children (ages 10–13) or the Perceived Competence Scale for Adolescents (ages 14–15) [33]. These scales are valid and reliable for the chosen population [34,35]. Participants responded to the physical competence subset consisting of six questions. The physical competence questionnaire consists of one example question and five assessment questions assessing how the children and adolescents feel regarding their ability to participate in physical activity (score of 1–4 per question; 1 being least confident and 4 being most confident). The mean score per question was used in the analysis.

#### 2.2.3. Physical Activity Assessment

Each participant wore an accelerometer (ActiGraph GT3X+, Pensacola, FL), which recorded physical activity during a seven-day period. The accelerometer was placed above the iliac crest of the right hip using a nylon elastic belt. The accelerometer was initialized to collect raw data at 30 Hz [36,37]. Physical activity data from accelerometers were recorded as raw activity counts, a measure reflecting the number of times raw acceleration data exceeded a pre-defined threshold. Activity count data were converted to minutes of physical activity using Evenson et al.’s [38] cut-points. Cut-points were used to classify the activity into four categories: sedentary (0–100 counts per minute or CPM); light (101–2295 CPM); moderate (2296–4011 CPM); and vigorous (4012 or more CPM). The total number of minutes of moderate and vigorous activity was then summed. Each participant’s total MVPA score was divided by the number of days during which the accelerometer was worn to yield a measure of average daily minutes of MVPA. Average daily MVPA minutes were used in the subsequent correlational analysis described below. Participants were excluded if they did not have at least three days (including one weekend day) of 10 h daily accelerometer wear time [39].

#### 2.2.4. Health-Related Fitness Assessment

Participants completed the FitnessGram Physical Fitness Battery to assess aerobic fitness, muscular strength, muscular endurance, flexibility, and body composition [40]. Only the Progressive Aerobic Cardiovascular Endurance Run (PACER; aerobic fitness), which is a paced, 20 m shuttle run was included in the analysis.

#### 2.2.5. Data Treatment and Statistical Analyses

A power analysis was conducted to estimate sample size using association studies (MC and PA, MC and fitness). A minimum sample of 44 was needed based on a significance of A-0.05 and a power of 0.80.

Demographic and anthropometric data included race, sex, age, and both standing and seated heights. BMI was calculated and BMI percentile was then identified using the sex-specific BMI-for-age percentile curves [41]. The predicted age of peak height velocity (PHV) was calculated from the participant’s birthdate, test date, standing height, sitting height, and weight using procedures described by Mirwald et al. [42]. A maturity offset score (i.e., the number of years away from predicted age of PHV) was then used to categorize each participant as pre-PHV, PHV, or post-PHV. Descriptive statistics and frequencies were calculated for demographic variables, BMI, weight status, and the pre-PVH maturity offset category.

Descriptive statistics were calculated for PMC for each visit and overall. A comparison of PMC scores from Visits 1 and 2 using independent *t*-tests showed no significant difference (*p* > 0.05); the higher of the two scores for each participant was used in subsequent analysis described below.

Descriptive statistics were calculated for all variables assessed. BMI data were not normally distributed, leading to nonparametric correlational analysis (Spearman’s rank-order correlations) being used to examine associations among MC, MVPA, PMC, aerobic fitness, and BMI.

Mediation and moderation models were analyzed using a bootstrapping approach to determine the indirect effects. MC was entered as the predictor variable with the outcome variable being MVPA. The proposed mediators in the model were PMC and physical fitness, with BMI as the proposed moderator. The PROCESS macro Model 4 (V.4.2) in SPSS was used to conduct these analyses; 95% confidence intervals were used to determine the significance of the indirect effects mediated by PMC and aerobic fitness and moderated by BMI. SPSS 27.0 (IBM, Armonk, NY, USA) was used for all statistical analyses. The alpha level was set to *p* < 0.05 to determine statistical significance.

## 3. Results

### Participant Characteristics

From the 47 participants (12.2 ± 1.6 y; 55% male) recruited for this study, 3 participants were excluded from the physical activity analyses because they did not meet wear time criteria. Table 1 shows the descriptive statistics of the participants. The prevalence of children with overweight/obesity in this sample was 27.7%. Most of the participants were classified as pre-PHV (89.4%), indicating that they had not started the maturation process. Most participants (72.3%) had average levels of motor competency. The average BOT-2 total motor composite score was 49.9 ± 9.0. Participants spent an average of 40.0 ± 17.6 min in MVPA per day, with only 16.7% of the participants meeting the aerobic physical activity guidelines. The mean PMC score was 3.1 ± 0.5 and the participants achieved a mean PACER score of 31.6 ± 14.9 laps.

Table 2 depicts the Spearman’s rank-order correlations for the total group. There were significant positive correlations between MC and aerobic fitness [*r_s_*(47) = 0.469, *p* < 0.01], PMC and aerobic fitness [*r_s_*(47) = 0.682, *p* < 0.01], and PMC and MC [*r_s_*(47) = 0.416, *p* < 0.01]. There were no significant associations between average daily MVPA and MC, PMC, or aerobic fitness (*p* > 0.05). There was a significant inverse association between BMI and both MVPA [*r_s_*(44) = −0.410, *p* < 0.01] and aerobic fitness [*r_s_*(47) = −0.295, *p* < 0.05].

**Figure 1:**
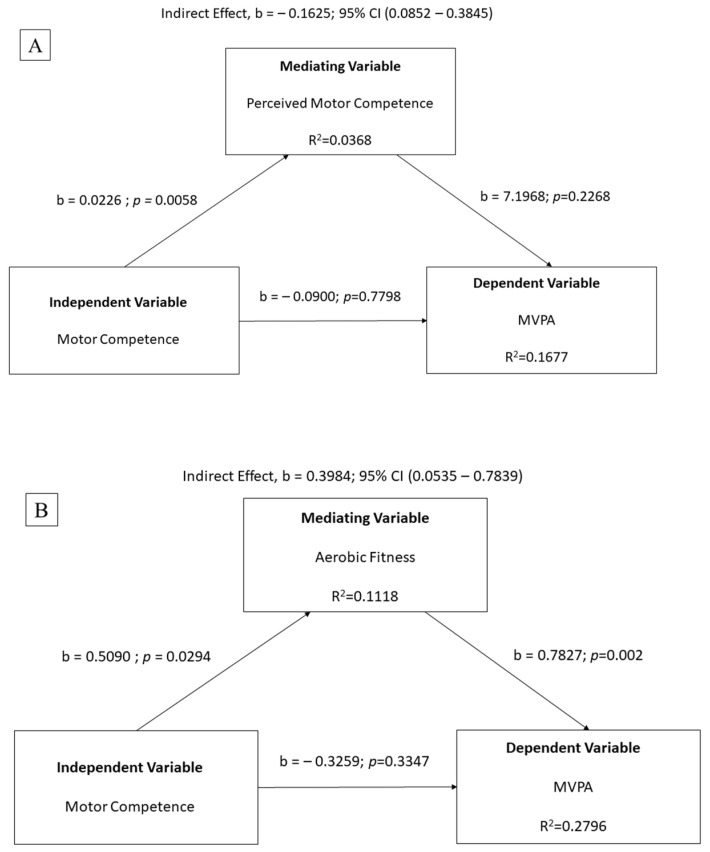
Mediating effects of (**A**) perceived motor competence and (**B**) aerobic fitness on the relationship between motor competence and moderate-to-vigorous physical activity (MVPA).

Figure 1 illustrates the results of the mediation analysis. There was no mediating effect of PMC on the MC–MVPA relationship (see Figure 1A). Aerobic fitness mediated the relationship between MC and MVPA (see Figure 1B). There was a significant indirect effect of MC on MVPA that was mediated by aerobic fitness [b = 0.3984; 95% CI (0.0535–0.7839)]. Additionally, aerobic fitness accounted for ~28% of the variance in the model. The was no significant moderating effect of BMI on the MC–MVPA relationship (r^2^ = 0.1534, F(1,40) = 0.2426, *p* = 0.6250).

## 4. Discussion

The purpose of this study was to determine the associations among MC, MVPA, PMC, and aerobic fitness in children and adolescents (10–15 years old). It was hypothesized that there would be significant, positive associations among all the variables (MC, MVPA, PMC, and aerobic fitness). Additionally, this study aimed to investigate the possible mediating (PMC and aerobic fitness) and moderating (BMI) effects on the MC–MVPA relationship. The current study provides an examination of the holistic framework of the proposed developmental model and includes a sample of youth (late childhood and adolescence) that have not been extensively studied compared to younger age groups. Additionally, the inclusion of the BOT-2 and the focus on MVPA might provide unique insights to further our understanding of the MC and physical activity relationship. While this study cannot imply causality, there are a few plausible explanations for why associations exist among some of these variables and the mediating effect of aerobic fitness.

There was a moderate, positive correlation between MC and PMC. The PMC scores were consistent with previously reported average ranges for children aged 10–15 years old [16]. Presumably, children with higher perceptions of their motor competence are more likely to engage in a greater variety of physical activities, further enhancing the development of their motor skills. In the present study, children had high perceptions of competence (average three out of four) and had above average MC rates as assessed by the BOT-2, which aligns with this notion; however, only 17% of children were meeting physical activity guidelines. It might be that the focus on MVPA (versus habitual physical activity) may have contributed to the mismatch among PMC and physical activity in the present study. Children with lower PMC scores are likely to participate in fewer physical activities, which would limit their opportunities to further develop their motor skills [10,43,44]. It is also likely that a child’s level of MC contributes to PMC, creating reciprocal reinforcement. The utilization of the PMC and MC assessments in the current study may have also contributed to the significant associations. Harter’s Perceived Competence Scales for children and adolescents assess perceptions of global physical competence and the BOT-2 measures a wide range of motor skills (fine and gross) in addition to coordination, strength, and agility, which may be viewed as a more global assessment of MC.

Another possibility is that one or more other variables affect both MC and PMC. A likely candidate would be aerobic fitness, since it was found to be positively correlated to both. These results suggest that promoting PMC may be an effective means to foster physical activity in middle childhood and adolescence. Although changes in MC and aerobic fitness can require extended periods of time to achieve noticeable results, readily available psychological interventions to increase PMC through techniques such as encouragement, goal setting, and reframing may allow practitioners to quickly increase the odds of a child engaging in physical activity [45]. This reasoning is consistent with previous research arguing that PMC can have a positive impact on adolescent’s MC [10,43,44,46,47] and that some children lack the motivation to participate in physical activity due to the belief that they are not able to be successful during activity engagement [47].

Research has shown that positive encouragement during physical and motor activities can lead to an increased PMC and promotion of other psychological factors that support activity engagement [43]. Additionally, a recent study proposed that psychological factors, such as PMC and intrinsic motivation, play a significant role in physical activity participation in youth [48]. Enjoyment is one of the main factors contributing to whether an individual will participate in an activity [47]. Additionally, higher levels of PMC may increase enjoyment of physical activity, which can help a child persist in their engagement of motor practice and improve their motor skills [46]. In addition, social interaction with peers is one way to simultaneously increase PMC and promote positive psychological benefits [47].

MC and aerobic fitness were also positively correlated. It appears that future physical fitness can be predicted by the level of gross motor skills during early childhood [49]. Fundamental motor skills are the foundation for the more complex motor skills required to improve aerobic fitness (i.e., most locomotor activities) [24,48,50]. These findings may provide an explanation for the significant association between MC and aerobic fitness, based upon running (PACER) performance [24,48,50]. In the present study, the full BOT-2 was used to measure MC and it includes a subscale which measures strength and agility, which aligns with the aerobic fitness results of the PACER test as children scored at or above average in both assessments. Presumably, exposure to similar movements, such as galloping and jumping, would enhance the neuromuscular system function as well as motor control and coordination [24]. Higher fitness levels also promote the attainment of motor competency throughout adolescence [51]. Additionally, youth in late childhood who are proficient in their motor skills tend to be classified in a healthy weight category. This may be due to the greater opportunity for activity participation and the ability to achieve success during these activities [51].

The mediation analysis of the current study revealed a significant, indirect effect of aerobic fitness on the MC and physical activity relationship. Stodden’s model postulated that fitness may mediate the relationship between MC and physical activity through the potential increase in engagement in physical activity in youth with higher fitness levels [10]. Numerous studies have identified aerobic fitness as a mediating variable or have suggested the exploration of the mediating impact of aerobic fitness [14,15]. Several studies have found fitness to have a significant mediating effect, but the majority of these studies focused on middle childhood [20,21,26]. The mediating effect of aerobic fitness provides some insight that demonstrates that youth may need to attain higher fitness levels to illustrate an influence of MC on physical activity levels. This study provides a significant contribution to the literature through the investigation of the mediating effect of fitness on youth in late childhood and adolescent age groups.

Findings from the current study indicate no significant associations among average daily MVPA and MC, PMC, or aerobic fitness. Only 17% of children in the present study met physical activity recommendations; this prevalence of meeting recommendations was lower compared to a nationally representative sample [52]. The results did not support the theory that older children’s motor skills are associated with their MVPA level. This is a different result than what was hypothesized by Stodden et al. regarding a positive relationship between MC and physical activity in this age group [10]. However, the original model proposed the relationship between total physical activity and not specifically MVPA. Higher amounts of MVPA are associated with a lower cardiovascular disease risk and tend to be associated with a higher cardiorespiratory fitness. The present study focused on health-enhancing MVPA levels in this age group, but this may have limited the ability to fully examine the relationship between MC and different intensities, including light PA, which has recently garnered more attention in terms of reducing overall sedentary behavior across a 24 h period. Research has shown significant positive associations among MC and physical activity in younger children (<10 years of age), but not older children (≥10 years of age) [10,17]. If children are not physically active at a young age, then their motor competency may not be established or progress appropriately [11,12]. Deficiencies in MC may also result from insufficient exposure to motor skills or opportunities to learn or practice motor skills (i.e., in physical education, sports teams, activity classes). Even if a group of adolescents (10–15 years old) are physically active, variations in their levels of motor skill proficiency developed during childhood would presumably decrease the chances of detecting a correlation between MC and MVPA. This would be compounded by the fact that MVPA cut-points can create homogenous groups in terms of activity counts, even when the sample includes a wide range of skill proficiencies for activities that may or may not contribute to assessments via accelerometers.

Differences in sport-specific skill requirements may allow some adolescents to achieve relatively high activity counts (i.e., MVPA) despite underdeveloped skills for a range of complex movements. For example, an adolescent could potentially compete in a sport such as cross-country running while not meeting age-related skill proficiency expectations for sports requiring object control skills such as throwing, kicking, and catching. In contrast, a basketball player would be likely to achieve high activity counts and possess a broader skill set because of the demands of the sport. Therefore, exposure to complex movement activities such as basketball during childhood might be expected to provide a stronger foundation for successfully participating in a broader range of physical activity during adolescence.

Overall, there are many strengths to this study. First, the sample size overall achieved an adequate power, based upon a power analysis that identified a target sample size of 44, and the sample was of equal proportion between males (54.3%) and females (45.7%). Also, validated, objective tools were used for all the assessments (BOT-2, Actigraph GT3X accelerometer, PMC, FitnessGram). This study also had several limitations. One limitation of this study is the homogenous sample. The sample only included Caucasian participants and it was a convenience sample, which may have caused selection bias; therefore, generalizations of these findings should be interpreted with caution. In addition, this population had a similar aerobic fitness level, but were less physically active compared to national samples [49,53]. This may be due to the accelerometers not capturing all of the MVPA that the youth participated in (such as bicycling or water-based activities). Anecdotally, some participants reported that they did not wear the accelerometer during certain sports and activities (i.e., baseball, swimming).

## 5. Conclusions

The main objective of the study was to assess the associations among MC, MVPA, PMC, and aerobic fitness in youth aged 10–15 years old and to explore the possible mediating and moderating factors impacting the relationship between MC and physical activity. There were significant associations among MC and PMC, MC and aerobic fitness, and PMC and aerobic fitness. In the current study, MVPA was not significantly associated with any of the other variables assessed. Therefore, this study only partially supports the developmental model concerning the relationships among MC, MVPA, PMC, and aerobic fitness [10]. One of this study’s main contributions to the current literature is finding that in children aged 10–15, MC is not associated with their engagement in MVPA, but there were significant indirect associations when considering the mediating influence of aerobic physical fitness. These findings build on previous research and provide further evidence for the proposed developmental trajectory model and for how these important factors influence adolescent health.

## Figures and Tables

**Table 1:** Descriptive statistics of the participants (n = 47).

Variable	Mean (SD)	*n* (%)
**Age (y)**	12.2 (1.7)	
**Mass (kg)**	50.5 (16.2)	
**Height (cm)**	157.2 (13.1)	
**BMI (kg/m^2^)**	20.0 (4.2)	
**Weight Status**		
Underweight		1 (2.1)
Healthy Weight		33 (70.2)
Overweight		10 (21.3)
Obese		3 (6.4)
**Maturity Offset**		
Pre-Peak Height Velocity		42 (89.4)
At-Peak Height Velocity		3 (6.4)
Post-Peak Height Velocity		2 (4.3)
**Sex**		
Male		26 (55.3)
Female		21 (44.7)
**Race**		
Caucasian		47 (100)

**Table 2:** Spearman rank-order correlation coefficients (*r_s_*).

Variable	Aerobic Capacity	Motor Competence	Perceived Motor Competence	MVPA
BMI	−0.295 *	−0.207	−0.318 *	−0.358 *
Aerobic Capacity		0.469 **	0.682 **	0.243
Motor Competence			0.416 **	−0.012
Perceived Motor Competence				0.212

Note: * Denotes significant correlation (*p* < 0.05, two-tailed); ** Denotes significant correlation (*p* < 0.01, two-tailed). BMI = body mass index; aerobic capacity measured by the PACER; motor competence measured by the BOT-2; perceived motor competence measured by Harter’s self-competency scales.

## Data Availability

The data presented in the current study are available upon request from the corresponding author. The data are not publicly available due to privacy and ethical restrictions.
